# Evaluation of Hospital Applications of Former Earthquake Victims Due to Re-Traumatization Through Media

**DOI:** 10.1192/j.eurpsy.2025.907

**Published:** 2025-08-26

**Authors:** H. O. Torun, B. Akdöner, T. Sarıkavak

**Affiliations:** 1 Psychiatry, İzmir City Hospital, İzmir; 2 Psychiatry, Near East University, Girne; 3 Psychiatry, Atlas University, İstanbul, Türkiye

## Abstract

**Introduction:**

The 2020 Aegean Sea earthquake occurred on October 30, 2020, 23 km from the Seferihisar district of Izmir, with a magnitude of 6.9. It caused the death of a total of 119 people and the injury of 1053 people in Turkey and Greece. After this earthquake, many people were observed to have symptoms of post-traumatic stress disorder and received treatment. The two major earthquakes centered in Kahramanmaraş on February 6, 2023 also caused great destruction in Turkey. After this earthquake, an increase in the mental complaints of people who had previously experienced the earthquake in Izmir Seferihisar was observed and these people applied to the psychiatry outpatient clinic.

**Objectives:**

Revealing how much former earthquake victims are affected by similar events through the media.

Determining the situations that cause people to be re-traumatized.

Observing the effects of the media on mental health.

**Methods:**

This study investigated the effects of former earthquake victims who applied to Izmir Seferihisar State Hospital after these new earthquakes, and who were not in the earthquake region at the time of the earthquake and who did not have any losses or injuries to relatives or acquaintances, through the media. For this purpose, after the earthquake centered in Kahramanmaraş, the Adult Resilience Scale and the Post-Traumatic Stress Disorder Checklist for DSM-5 were applied to these individuals at their first application, and it was questioned whether the individuals had received pharmacotherapy and psychotherapy after the previous Aegean Sea earthquake. It was also investigated how the individuals followed the news about the new earthquake. Afterwards, whether the individuals received treatment and the duration of this treatment were recorded, and the Post-Traumatic Stress Disorder Checklist for DSM-5 was applied to the individuals at the 1st, 3rd, 6th, 9th and 12th months.

**Results:**

People who have received psychotherapy are 1.9 times less likely to need treatment afterwards.

Women are 4.1 times more likely to be affected by the media and use SSRIs.

People who have lost their homes need treatment as often as those whose close relatives have died.

The risk of people who do not receive treatment after a disaster being affected by the media and receiving treatment is 3.4 times higher than those who received treatment during the first disaster.

**Conclusions:**

After disasters, when another disaster occurs, PTSD symptoms can be observed again in former disaster victims. Post-traumatic psychotherapy can also be protective in terms of future situations. Watching traumatic events in the media can cause PTSD symptoms to be seen even if no relatives have been harmed.

**Disclosure of Interest:**

None Declared

